# UPrimer: A Clade-Specific Primer Design Program Based on Nested-PCR Strategy and Its Applications in Amplicon Capture Phylogenomics

**DOI:** 10.1093/molbev/msad230

**Published:** 2023-10-13

**Authors:** JiaXuan Li, GuangCheng Han, Xiao Tian, Dan Liang, Peng Zhang

**Affiliations:** State Key Laboratory of Biocontrol, School of Life Sciences, Sun Yat-Sen University, Guangzhou 510275, China; State Key Laboratory of Biocontrol, School of Life Sciences, Sun Yat-Sen University, Guangzhou 510275, China; State Key Laboratory of Biocontrol, School of Life Sciences, Sun Yat-Sen University, Guangzhou 510275, China; State Key Laboratory of Biocontrol, School of Life Sciences, Sun Yat-Sen University, Guangzhou 510275, China; State Key Laboratory of Biocontrol, School of Life Sciences, Sun Yat-Sen University, Guangzhou 510275, China

**Keywords:** nuclear protein-coding locus, primer design, amplicon capture, homemade probes, phylogenomics

## Abstract

Amplicon capture is a promising target sequence capture approach for phylogenomic analyses, and the design of clade-specific nuclear protein-coding locus (NPCL) amplification primers is crucial for its successful application. In this study, we developed a primer design program called UPrimer that can quickly design clade-specific NPCL amplification primers based on genome data, without requiring manual intervention. Unlike other available primer design programs, UPrimer uses a nested-PCR strategy that greatly improves the amplification success rate of the designed primers. We examined all available metazoan genome data deposited in NCBI and developed NPCL primer sets for 21 metazoan groups with UPrimer, covering a wide range of taxa, including arthropods, mollusks, cnidarians, echinoderms, and vertebrates. On average, each clade-specific NPCL primer set comprises ∼1,000 NPCLs. PCR amplification tests were performed in 6 metazoan groups, and the developed primers showed a PCR success rate exceeding 95%. Furthermore, we demonstrated a phylogenetic case study in Lepidoptera, showing how NPCL primers can be used for phylogenomic analyses with amplicon capture. Our results indicated that using 100 NPCL probes recovered robust high-level phylogenetic relationships among butterflies, highlighting the utility of the newly designed NPCL primer sets for phylogenetic studies. We anticipate that the automated tool UPrimer and the developed NPCL primer sets for 21 metazoan groups will enable researchers to obtain phylogenomic data more efficiently and cost-effectively and accelerate the resolution of various parts of the Tree of Life.

## Introduction

Target sequence capture is a powerful high-throughput sequencing technique that has revolutionized evolutionary biology research in recent years (Lemmon and Lemmon 2013; Jones and Good 2016). This technique uses capture probes, also known as baits, to selectively enrich genomic regions of interest, allowing for efficient and cost-effective sequencing of large numbers of genomic loci across a diverse range of taxa. In the field of phylogenetics, researchers can use probes designed to capture specific genomic regions of interest to obtain high-quality data from large numbers of samples. In addition, this approach has low DNA quality requirements and can analyze samples with serious DNA degradation (e.g. Guschanski et al. 2013; Blaimer et al. 2016). Consequently, target sequence capture has become a widely used sequencing method in phylogenetic research, enabling researchers to uncover the evolutionary history of a wide range of organisms from plants to animals and promising to continue driving discoveries in this field in the future (e.g. Prum et al. 2015; Léveillé-Bourret et al. 2018; Xu et al. 2021).

Currently, anchored hybrid enrichment (AHE) sequencing (Lemmon et al. 2012) and ultraconserved element (UCE) sequencing (Faircloth et al. 2012) are the 2 most widely used target sequence capture methods in phylogenetic research. Both approaches rely on commercially synthesized DNA/RNA probes to capture short, highly conserved regions in the genome. However, using commercial probes can be prohibitively expensive when dealing with hundreds to thousands of samples. Additionally, predesigned commercial probes may not be available for many nonmodel organism groups. Therefore, developing alternative target sequence capture methods without using commercial probes is highly valuable.

**Fig. 1.**
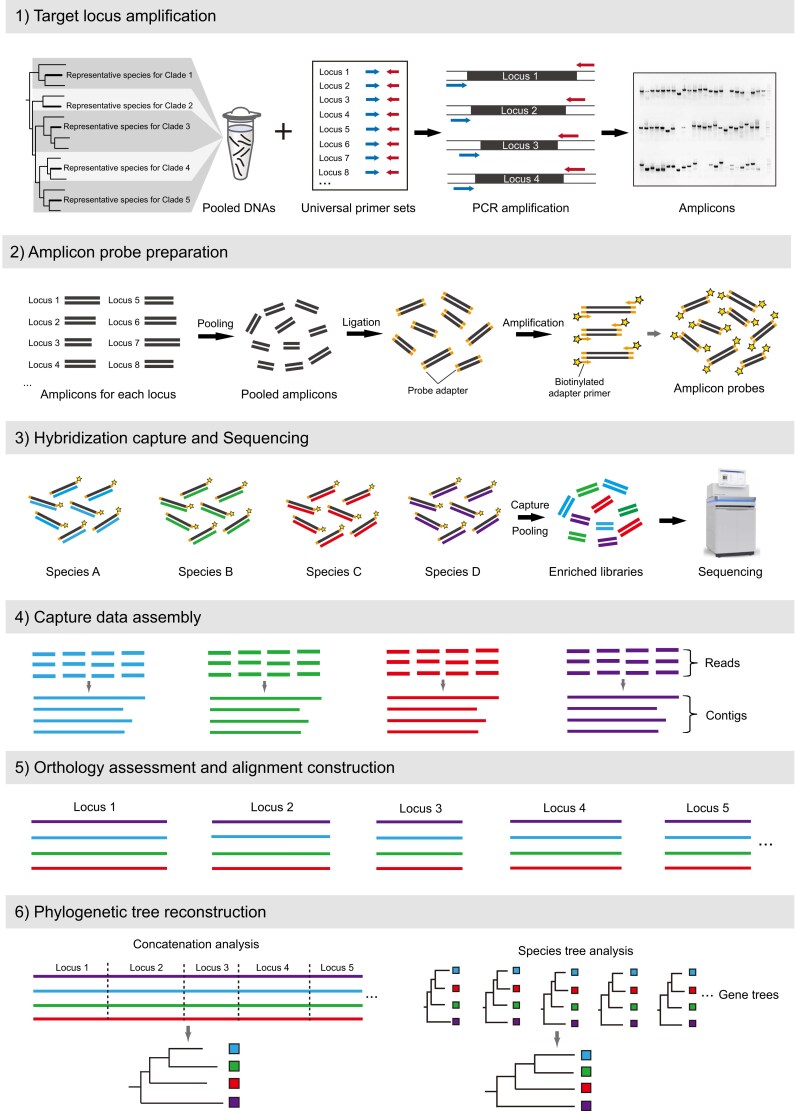
General workflow for implementing amplicon capture in phylogenomics.

Recently, some research teams have reported that biotinylated amplicons can serve as capture probes for target sequence capture, a strategy that can be referred to as “amplicon capture” (Peñalba et al. 2014; Knyshov et al. 2019; Zhang, Deng, et al. 2019). Nuclear protein-coding loci (NPCLs) are ideal target capture loci for amplicon capture because they can be amplified using universal primers across taxa of interest, are abundant in the genome (Wild and Maddison 2008), and exhibit an appropriate degree of conservation across a wide phylogenetic range (Thomson et al. 2010; Winkler et al. 2015). The workflow of amplicon capture is illustrated in Fig. 1. The most critical step of this approach is using universal primers to amplify a bulk of target NPCLs from some representative species of the organism group under study. A biotinylated adapter is then added at both ends of these amplicons to generate amplicon probes. Because the amplicons amplified from the representative species have high sequence similarity to the target regions of the other species of the studied organism group, they can be used as probes for target sequence capture. Compared with AHE or UCE sequencing based on commercial probes, amplicon capture enables researchers to create their own capture probes in the lab, with high flexibility in probe preparation, as the number and composition of amplicons can be adjusted according to the project's needs at any time (Knyshov et al. 2019; Zhang, Ding, et al. 2019; Zhang, Deng, et al. 2019). In addition, the NPCLs obtained by amplicon capture are generally longer than those loci produced by UCE or AHE probes. Longer sequence length can reduce gene tree estimation error (GTEE) and lead to more accurate species tree estimation (Salichos and Rokas 2013; Roch and Warnow 2015; Karin et al. 2020). Finally, the probe cost per sample for amplicon capture is 2 to 10 times lower than that of capture methods based on commercial probes (depending on the actual number of probes used), making it particularly suitable for projects with a large number of samples.

To ensure successful amplicon capture, it is crucial to have universal amplification primers that can effectively amplify target NPCLs across a wide phylogenetic range. However, despite previous efforts to develop NPCL primer sets for several animal groups, including ray-finned fishes (Li et al. 2007), vertebrates (Shen et al. 2013), birds (Kerr et al. 2014; Liu et al. 2018), beetles (Che et al. 2017), and lepidopterans (Zhang, Deng, et al. 2019), many animal groups, particularly invertebrates, still lack such primer sets. Furthermore, currently available NPCL primer sets typically target a limited number of NPCLs (usually ≤100). However, this quantity of loci may be insufficient for a standard target sequence capture experiment. Therefore, to facilitate wider application of amplicon capture technology, it is necessary to develop larger-scale NPCL primer sets for various animal lineages, especially those invertebrate groups with less studied phylogenies.

Traditionally, developing NPCL amplification primers from genomic data involves multiple manual steps. Researchers must search for suitable coding regions in genomes, construct homologous multiple sequence alignments (MSAs), manually check hundreds to thousands of MSAs, identify conserved priming sites, optimize primer design, and consider target amplification region variability. This process is highly time-consuming and requires extensive experience. To improve efficiency, programs such as MarkerMiner (Chamala et al. 2015) and DOMINO (Frías-López et al. 2016) have been developed to assist researchers in accelerating the development process. These programs can process transcriptome and genome data to identify suitable loci and output MSAs for primer development. Other programs, such as ecoPrimers (Riaz et al. 2011), PolyMarker (Ramirez-Gonzalez et al. 2015), and DISCOMARK (Rutschmann et al. 2017), can design primers from precomputed MSAs. However, no program currently exists that can simultaneously identify suitable gene loci, create MSAs from genomic data, and design primers. Moreover, previous primer design programs use standard-PCR amplification strategy (i.e. designing 1 primer pair to amplify a target region), which generally has a low success rate. Our previous study has shown that nested PCR, which uses 2 pairs of nested-PCR primers to amplify a target region, is more effective in amplifying NPCLs (Shen et al. 2012). We argue that developing a primer design program capable of designing nested-PCR primers for NPCLs may solve the problem of low PCR success rate.

In the postgenomic era, the increasing amount of genomic data has provided a solid foundation for the development of NPCL primer sets for different animal groups. As of the time of writing, the NCBI genome database had contained more than 4,500 animal genomes, encompassing most of the major metazoan groups, including cnidarians, flatworms, nematodes, annelids, arthropods, mollusks, echinoderms, and vertebrates. However, manually analyzing these genome data to develop NPCL primer sets is nearly impossible. What we need is an easy-to-use bioinformatics tool that can utilize genomic data and automate the entire process of developing NPCL primer sets with a high success rate of amplification.

To achieve this objective, we have developed a program called UPrimer, which utilizes genome data of a target organism group to automatically design clade-specific NPCL amplification primers, with minimal manual involvement. UPrimer adopts a nested-PCR amplification strategy to design NPCL primers, which significantly increases the success rate of gene amplification. With just a single command line, UPrimer can identify hundreds to thousands of NPCLs and generate universal primer sets for them, making it user-friendly for researchers without prior experience in genome data processing and primer design. We systematically investigated the metazoan genome data deposited in the NCBI database and used UPrimer to develop NPCL primer sets for dozens of metazoan groups, which covered a diverse range of invertebrate and vertebrate groups. We also tested the success rate of gene amplification using UPrimer-developed NPCL primers in 6 metazoan groups. Finally, we conducted a phylogenetic analysis by sequencing 100 NPCLs for 26 butterfly and 8 moth species using amplicon capture, and successfully reconstructed a robust high-level phylogenetic relationship of butterflies.

## Materials and Methods

### The Design Architecture of UPrimer

**Fig. 2.**
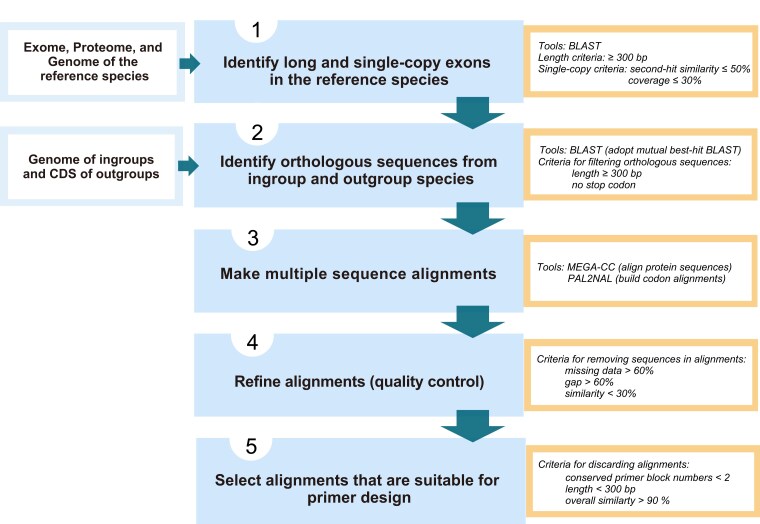
Flowchart illustrating the first module of UPrimer, aiming at generating candidate MSAs for primer design, utilizing genomic data from the taxonomic group under investigation.

The whole workflow of UPrimer comprises 2 main modules. The first module aims to obtain candidate MSAs based on the genome data of the target taxon (Fig. 2). The module contains 5 main steps:

1. Identify long and single-copy exons from the genome of a reference species. The reference species can be any species of the target taxon but should have well-annotated genome data available. The input data of this step are exome, proteome, and genome sequences of the reference species. UPrimer first uses BLASTX to trim each exon in the exome to the correct translation frame using the proteome as a guide. Subsequently, it discards exons shorter than a predefined value (default: 300 bp). The program then uses BLASTN to search the remaining exons against the genome to remove exons that are not single copy. The criterion is as followed: if an exon has a second BLAST hit with similarity > 50% and coverage > 30%, this exon is considered to have a similar copy in the genome and is not single copy.
2. Obtain orthologous sequences of the exons of the reference species from ingroup and outgroup species. The ingroup species (required) belong to the same target taxon as the reference species, and it is better to use more ingroup species to cover the whole phylogenetic span of the target taxon. Outgroup species (optional) do not belong to the target taxon, and having more outgroup species in the analysis can ensure finding conserved regions for primer design. The input data of this step are genome sequences of the ingroup species and coding sequences (CDSs or transcriptome) of outgroup species. Based on the exons of the reference species, UPrimer employs a mutual best-hit blast strategy (MBH BLAST) to extract orthologous sequences from the genomes or CDSs of both ingroup and outgroup species. Among the identified orthologous sequences, only those with a length greater than 300 bp and without stop codons are retained. For each exon of the reference species, the program combines all its filtered orthologous sequences from both ingroup and outgroup species, constructing orthologous sequence groups (OGs) at both the DNA and protein levels.
3. Construct MSAs for each OG. UPrimer first aligns the OGs’ protein sequences using MEGA-CC (Kumar et al. 2012). Subsequently, PAL2NAL (Suyama et al. 2006) is utilized to construct codon alignments by incorporating the DNA sequences of the OGs and the resulting protein alignments. The program then trims the protein and DNA alignments on both ends, guided by the exons of the reference species.
4. Remove problematic sequences and trim alignments to increase the quality of MSAs. UPrimer will check each MSA and discard sequences that have high levels of missing data (>60% of *N*/gaps). Furthermore, in order to eliminate problematic sequences in a MSA resulting from incorrect orthology assignment or sequence errors, if a sequence's average similarity to all other sequences within the alignments falls below 30%, it will also be discarded. The 30% cutoff value is the default setting and can be adjusted according to the experience of users.
5. Pick out suitable MSAs for subsequent primer design. After refining the alignments, UPrimer proceeds to select suitable candidate alignments for primer design. It first searches each alignment from both the left and right ends to ensure the presence of 2 conserved primer blocks, each consisting of 8 amino acids and exhibiting a sequence similarity greater than 50%. Subsequently, the alignment is trimmed by removing the regions outside the leftmost and rightmost primer blocks. The trimmed alignments must have a length exceeding 300 bp; otherwise, they are discarded. Additionally, UPrimer will discard highly conserved alignments (with a similarity greater than 90%) that contain too few informative sites.

**Fig. 3.**
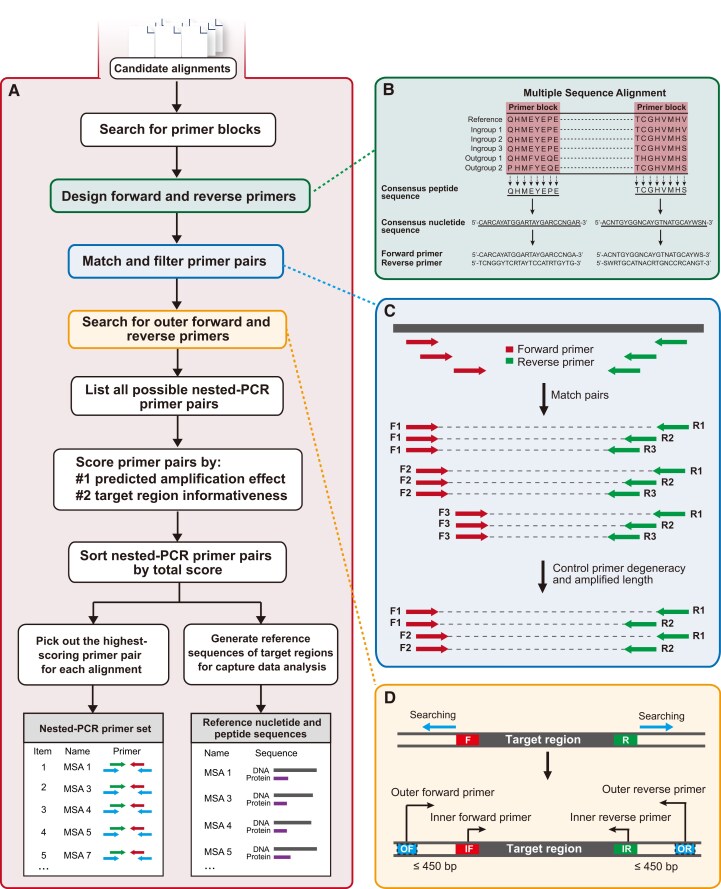
a) Schematic overview of the second module of UPrimer for developing nested-PCR primers for NPCLs using candidate alignments. The output includes a table of NPCL primers for target locus collection and amplicon probe preparation, as well as reference nucleotide and peptide sequences for capture data analysis. b–d) illustrate the detailed processes of primer design, primer matching and filtering, and outer primer searching, respectively.

The second module aims to design universal nested-PCR primer sets of NPCLs based on candidate MSAs. The workflow of this module is illustrated in Fig. 3a. For each candidate MSA, the program first searches for all conserved primer blocks that are 7 or 8 amino acids in length and subsequently designs forward and reverse primers from these identified blocks (Fig. 3b). Next, the program matches all forward and reverse primers to list all possible primer pairs and filters them by primer degeneracy and amplification length (Fig. 3c). For every retained primer pair, UPrimer searches for their outer forward and reverse primers (if they exist) within a flanking region of 450 bp (Fig. 3d), obtaining a list of all possible nested-PCR primer pairs. Then, the program scores each nested-PCR primer pair based on its potential PCR performance (named “ScorePCR”), taking into account the conservation of primer blocks, primer degeneracy, and primer complexity, as well as the phylogenetic informativeness of the locus (named “ScoreINFOR”), which depends on the variability of the amplification regions (detailed calculation algorithm can be found at https://github.com/zhangpenglab/UPrimer#scoring-algorithms-for-primers). The program then calculates a total score for each primer pair, based on a weighting parameter “PIs” (default = 1) of the 2 scores. The formula for calculation is as follows:


$$\mathrm{Total}\mathrm{score}=\frac{(\mathrm{PIs}*\mathrm{ScorePCR})+\mathrm{ScoreINFOR}}{1+\mathrm{PIs}\;}.$$

Finally, the primer pairs are sorted by total score, and the highest-scoring primer pair is selected from the primer list of each MSA. The final output includes a nested-PCR primer table, as well as the reference nucleotide and peptide sequences of the NPCL regions, which will be used for subsequent capture data analysis.

### Developing NPCL Primer Sets for Different Metazoan Groups with UPrimer

We first conducted a survey of metazoan genome data available in the NCBI genome database up to June 2022 and found 4,557 species with genome sequences. The purpose of the survey was to identify metazoan groups with sufficient genome data to develop NPCL primer sets using UPrimer. In this context, a metazoan group refers to a particular phylum, subphylum, class, order, or suborder of Metazoa. Our survey identified 21 metazoan groups that had adequate genome data to develop NPCL primer sets, which included 1 phylum (Echinodermata), 1 subphylum (Vertebrata), 4 classes (Bivalvia, Cephalopoda, Gastropoda, and Hexanauplia), 12 orders (Araneae, Sarcoptiformes, Decapoda, Entomobryomorpha, Blattaria, Lepidoptera, Coleoptera, Hymenoptera, Diptera, Actiniaria, Scleractinia, and Cyclophyllidea), and 3 suborders (Heteroptera, Sternorrhyncha, and Auchenorrhyncha).

For each metazoan group, we selected a species with high-quality genome assembly and annotation as reference and at least 5 species from different major clades of the target metazoan group as ingroups. Outgroup species were included whenever possible for each primer developing analysis, but some metazoan groups, such as Echinodermata and Vertebrata, lacked suitable outgroup genome data, resulting in no outgroups included in their analyses. For Lepidoptera and Heteroptera, we used 2 different reference species while maintaining the same ingroups and outgroups to assess the impact of changing reference species. The primer developing information for the 21 metazoan groups is presented in Table 1, including the taxonomic classification, the reference species used, and the number of ingroups and outgroups. Information of the genome resource, such as accession numbers, is provided in supplementary Table S1, Supplementary Material online.

**Table 1:** UPrimer’s development of NPCL primers for 21 metazoan groups

Phylum	Subphylum	Class	Order	Suborder	Ingroups	Outgroups	Reference species	Exons	Long and single copy exons	Candidate MSAs	Nested-PCR primer set of NPCLs		
											Amplicons	Mean length	Download
Arthropoda	Chelicerata	Arachnida	Araneae		10	5	*Parasteatoda tepidariorum*	306,646	16,568	2,466	678	828	Supplementary Appendix S1, Supplementary Material online
			Sarcoptiformes		12	5	*Dermatophagoides farinae*	51,809	18,121	4,844	1,399	736	Supplementary Appendix S2, Supplementary Material online
	Crustacea	Hexanauplia			8	4	*Eurytemora affinis*	326,230	16,205	1,388	413	800	Supplementary Appendix S3, Supplementary Material online
		Malacostraca	Decapoda		13	4	*Penaeus chinensis*	312,822	22,020	1,776	704	828	Supplementary Appendix S4, Supplementary Material online
	Hexapoda	Entognatha	Entomobryomorpha		6	4	*Folsomia candida*	306,942	37,247	4,163	475	556	Supplementary Appendix S5, Supplementary Material online
		Insecta	Blattaria		5	6	*Zootermopsis nevadensis*	282,956	21,744	3,754	1,161	746	Supplementary Appendix S6, Supplementary Material online
			Lepidoptera		11	5	*Bombyx mori*	267,888	19,948	3,034	1,048	763	Supplementary Appendix S7, Supplementary Material online
					11	5	*Danaus plexippus*	101,573	9,055	1,881	590	749	\
			Coleoptera		10	5	*Anoplophora glabripenni*	103,603	17,060	7,346	1,131	669	Supplementary Appendix S8, Supplementary Material online
			Hymenoptera		12	5	*Bombus terrestris*	91,302	26,220	6,691	1,090	726	Supplementary Appendix S9, Supplementary Material online
			Diptera		9	5	*Drosophila melanogaster*	81,501	37,130	7,830	2,132	682	Supplementary Appendix S10, Supplementary Material online
			Hemiptera	Heteroptera	10	4	*Cimex lectularius*	250,049	17,408	2,483	526	706	Supplementary Appendix S11, Supplementary Material online
					10	4	*Rhodnius prolixus*	89,655	6,403	1,309	328	706	\
				Sternorrhyncha	10	5	*Rhopalosiphum maidis*	174,469	15,930	2,824	648	688	Supplementary Appendices S12– S21, Supplementary Material online
				Auchenorrhyncha	6	4	*Homalodisca vitripennis*	262,717	12,464	1,456	404	787	Supplementary Appendices S13–S21, Supplementary Material online
Mollusca		Bivalvia			10	5	*Crassostrea gigas*	699,754	42,765	3,507	690	695	Supplementary Appendix S14, Supplementary Material online
		Cephalopoda			6	4	*Octopus sinensis*	372,681	22,260	2,572	1,062	728	Supplementary Appendix S15, Supplementary Material online
		Gastropoda			12	5	*Gigantopelta aegis*	318,145	20,537	3,511	685	677	Supplementary Appendix S16, Supplementary Material online
Cnidaria	Actinozoa	Hexacorallia	Actiniaria		6	4	*Actinia tenebrosa*	232,778	23,389	6,328	1,981	677	Supplementary Appendix S17, Supplementary Material online
			Scleractinia		7	4	*Stylophora pistillata*	260,294	23,567	6,327	2,316	667	Supplementary Appendix S18, Supplementary Material online
Platyhelminthes	Eucestoda	Cestoda	Cyclophyllidea		7	6	*Echinococcus granulosus*	75,264	12,363	5,237	1,035	654	Supplementary Appendix S19, Supplementary Material online
Echinodermata					11	0	*Asterias rubens*	273,448	18,845	5,772	934	754	Supplementary Appendix S20, Supplementary Material online
Chordata	Vertebrata				8	0	*Xenopus tropicalis*	588,970	27,669	5,523	1,043	760	Supplementary Appendix S21, Supplementary Material online

### PCR Amplification Tests of the Newly Designed Primer Sets

To evaluate the PCR performance of the primer sets designed by UPrimer, we performed amplification tests on 6 representative metazoan groups: Decapoda, Gastropoda, Vertebrata, Araneae, Heteroptera, and Lepidoptera. For each group, we selected the top 60 primer pairs with the highest scores in their respective primer tables generated by UPrimer, except for Lepidoptera, where we randomly selected 100 primer pairs. The Lepidoptera primer pairs were not only assessed for their PCR performance but also evaluated for their phylogenetic utility through amplicon capture. The tested PCR primers of 6 metazoan groups can be found in supplementary Table S2, Supplementary Material online.

Genomic DNAs of *Eriocheir sinensis* (Decapoda, Varunidae), *Lissachatina fulica* (Gastropoda, Achatinidae), *Xenopeltis hainanensis* (Vertebrata, Xenopeltidae), *Tetragnatha nitens* (Araneae, Araneidae), *Coranus* sp. (Heteroptera, Reduviidae), and *Euthalia yasuyukii* (Lepidoptera, Nymphalidae) were used as DNA templates. The PCR program employed for all PCR reactions was uniform, consisting of an initial denaturation at 94 °C for 4 min, followed by 35 cycles of 94 °C for 45 s, 50 °C for 40 s, 72 °C for 2 min, and a final extension at 72 °C for 10 min. For the nested PCR, the first-round PCR was conducted in a 25-µL reaction mixture containing 10 ng of DNA template, 1× PCR buffer, 200 µM dNTPs, 400 nM of each forward and reverse first-round primers (referred to as outer primer pairs in Fig. 3d), and 1.25 U of Taq polymerase (TransTaq High Fidelity; TransGen, Beijing). The second-round PCR was performed in a similar manner to the first-round PCR, with the exception of using the second-round primers (also referred to as inner primer pairs in Fig. 3d) and 1 µL of the first-round PCR product as the DNA template. In order to compare the amplification efficiency between nested PCR and standard PCR, we also employed a standard-PCR strategy to amplify the target NPCLs using the second-round primers and animal DNA template directly. Finally, both the nested-PCR and standard-PCR products were analyzed on a 1.0% TAE agarose gel to verify their amplification.

### Demonstration of Amplicon Capture in Lepidoptera Phylogenetics

#### Taxon Sampling, DNA Extraction, and Library Preparation

As a demonstration, we conducted a phylogenetic analysis of Lepidoptera using amplicon capture. We sampled a total of 33 lepidopteran species, covering 6 butterfly families (Papilionidae, Pieridae, Lycaenidae, Hesperiidae, Riodinidae, and Nymphalidae) as ingroups and 7 moth families (Pyralidae, Drepanidae, Endromidae, Lasiocampidae, Erebidae, Sphingidae, and Noctuidae) as outgroups. Detailed information on these samples, such as taxonomy, collection locality, and voucher ID, is provided in supplementary Table S3, Supplementary Material online.

For each sample, genomic DNA was extracted from 1 to 3 legs using the TIANamp Genomic DNA Kit (TIANGEN Inc., Beijing, China). All DNA extracts were quantified using an ND-2000 spectrophotometer and diluted to a concentration of 10 ng/µL with 1× TE buffer. One hundred nanograms of each DNA sample were sheared to a size of 300 to 550 bp using Scientz18-A ultrasonic processors (SCIENTZ, Zhejiang Province, China). The fragmented DNA was used for Illumina library preparation with the NEBNext Ultra DNA Library Prep Kit (New England Biolabs Inc.). Each sample was labeled with a unique 8-bp index sequence. Three or four libraries were mixed into a pooled library in equal concentrations for subsequent hybridization capture.

#### Amplicon Probe Preparation, Hybridization, and Sequencing

Our amplicon capture targets include a total of 100 NPCLs of Lepidoptera. To generate amplicon probes, we followed the method of Zhang, Deng, et al. (2019) with some modifications. First, we mixed 10 µL of DNA extract from each of the 13 selected lepidopteran samples (indicated in Table 2) to make a DNA pool, which was subsequently used as DNA template to amplify the 100 NPCLs using the 100 lepidopteran nested-PCR primer pairs tested previously. The 100 PCR products were mixed in equal volume and purified using AMPure XP beads. The amplicon mixture was then 5′-phosphorylated and ligated with a BioT-linker at both ends, which was created by annealing 2 13-base oligos: 5′-Biotin-CAAGGACATCCGT-3′ and 5′-CGGATGTCCTTGC-3′. The linker-ligated amplicons were purified once again using AMPure XP beads and amplified with the 5′-biotinylated linker primer (Biotin-CAAGGACATCCGT) to generate amplicon probes. The PCR reaction mixture contained 1.25 U of HiFi Taq DNA Polymerase, 1× PCR buffer, 200 µM dNTP, 500 nM linker primer, and 100 ng of purified linker-ligated amplicons in a total volume of 25 µL. The thermal cycling program consisted of an initial denaturation for 30 s at 98 °C, followed by 10 cycles of 30 s at 94 °C, 45 s at 45 °C, and 2 min at 72 °C. The amplification product was purified with AMPure XP beads and quantified by ND-2000 spectrophotometer.

**Table 2:** Capture and sequencing results of the 33 lepidoptera samples

Family	Species	Clean reads	Assembled contigs	NPCL recovered	Nucleotide recovered (bp)	On target	Flanking exons (bp)
Papilionidae	*Papilio elwesi* ^a^	8,586,821	179,928	99	69,825	36.80%	52,044
	*Atrophaneura aidoneus*	5,679,546	11,736	99	65,538	46.56%	28,488
	*Parnassius epaphus*	8,683,918	55,310	99	65,139	35.03%	28,686
	*Bhutanitis thaidina*	19,880,711	136,792	99	67,620	46.60%	48,642
	*Sericinus montela*	4,424,705	25,784	99	65,652	57.01%	31,047
	*Luehdorfia puziloi*	4,451,761	28,835	96	55,917	55.58%	19,722
Hesperiidae	*Satarupa monbeigi*	7,465,486	214,284	100	69,840	39.96%	50,883
	*Choaspes benjaminii*	8,366,987	195,425	100	69,561	20.56%	40,302
	*Thymelicus leoninus* ^a^	12,328,903	450,470	100	71,133	32.90%	51,771
	*Carterocephalus stax*	10,898,891	148,659	100	70,389	44.20%	55,392
	*Celaenorrhinus dhanada*	10,248,278	9,122	99	69,327	21.68%	37,842
Pieridae	*Pieris canidia*	5,963,146	59,297	99	60,849	20.64%	31,155
	*Eurema hecabe* ^a^	3,894,540	60,206	99	67,572	39.89%	43,809
Riodinidae	*Takashia nana* ^a^	13,770,882	245,022	100	70,230	38.04%	55,245
	*Polycaena kansuensis*	21,678,279	182,880	99	67,971	31.56%	47,379
Lycaenidae	*Curetis acuta* ^a^	13,143,092	477,605	99	68,865	29.12%	51,420
	*Celastrina oreas*	4,935,650	119,131	100	69,348	28.71%	44,091
	*Taraka shiloi*	4,262,690	113,520	100	69,009	31.18%	48,150
	*Spalgis epius*	7,564,677	112,431	99	68,061	33.69%	52,902
Nymphalidae	*Euthalia yasuyukii* ^a^	5,149,426	127,607	99	68,838	51.08%	53,238
	*Stichophthalma howqua*	9,353,167	166,081	99	69,492	29.58%	52,101
	*Parantica aglea*	5,567,870	80,234	100	67,281	27.97%	41,136
	*Polyura narcaea*	11,034,817	216,228	99	67,830	33.76%	48,921
	*Kaniska canace*	16,346,266	571,529	99	69,081	37.85%	52,842
	*Libythea lepita*	12,838,394	443,652	99	67,116	30.12%	49,242
	*Ariadne merione*	7,986,346	150,057	100	69,687	45.71%	51,879
Drepanidae	*Cyclidia substigmaria* ^a^	9,134,876	338,859	100	70,713	34.21%	54,138
Endromidae	*Mustilia hepatica* ^a^	17,905,323	446,099	100	70,281	46.08%	55,353
Lasiocampidae	*Gastropacha pardale* ^a^	17,888,474	528,199	100	69,993	30.47%	50,628
Pyralidae	*Spoladea recurvalis* ^a^	11,135,659	405,057	100	68,583	39.43%	54,330
Erebidae	*Calliteara melli* ^a^	7,585,205	290,364	100	70,764	24.78%	53,934
Sphingidae	*Agrius convolvuli* ^a^	23,677,898	653,009	100	61,941	38.31%	46,110
Noctuidae	*Callopistria pulchrilinea* ^a^	12,429,784	412,179	100	68,880	39.22%	52,326

^a^Samples used to prepare amplicon probe.

For hybridization capture experiment, we followed a previously published capture protocol (Li et al. 2013) with some modifications. In each capture reaction, 500 ng of DNA libraries and 20 ng of amplicon probes were used. To enhance the capture efficiency, we implemented a touchdown hybridization program, which started with a denaturation step at 94 °C for 5 min, followed by hybridization at 65 °C, and then gradually decreasing the temperature by 5 °C every 6 h until it reached 45 °C, for a total of 30 h. The captured DNA fragments were isolated using streptavidin magnetic beads (Dynabeads MyOne bead, Life Technologies) and then washed to eliminate unhybridized DNAs. The captured DNA fragments were eluted with 30 µL 1× TE buffer and subsequently amplified using Illumina P5 and P7 universal primers. To increase the capture efficiency, we repeated the capture procedure using the enriched library obtained from the first hybridization and another 20 ng of amplicon probes. Finally, the second hybridization libraries from different capture experiments were pooled in equal concentrations and sequenced on 3 lanes of Illumina HiSeq X-ten with paired-end 150-bp mode.

#### NGS Data Processing

First, the Illumina paired-end reads were sorted into their respective species according to the 8-bp species index. Next, Trimmomatic v0.32 (Bolger et al. 2014) was used to filter out adapter sequences and low-quality nucleotides from the raw reads of each species. After filtering, the resulting clean reads for each species were assembled into contigs using metaSPAdes v3.13.0 (Nurk et al. 2017). The obtained contigs were then filtered for redundancy using CD-HIT-EST (Li and Godzik 2006) with a 95% similarity cutoff. To ensure high sequencing quality, only contigs with an average sequencing depth of ≥5× were retained for further analysis.

To extract the target NPCL sequences from the filtered contigs, the reference nucleotide and peptide sequences of the 100 NPCLs provided by UPrimer (Fig. 3a) were used as guide sequences. First, TBLASTN (*e* < 1e^−5^, identity > 50%; Boratyn et al. 2013) was performed to identify orthologous contigs based on the reference peptide sequences. Then, a reversed BLASTN (*e* < 1e^−5^, identity > 50%) was performed on the identified orthologous contigs against the reference nucleotide sequence to detect potential chimeras. As the orthologous contigs contained flanking sequences of the target regions, EXONERATE version 2.4.0 (Slater and Birney 2005) was employed to identify potential intron–exon boundaries based on the reference protein sequence of each target NPCL. All these steps were executed using a custom Python script called “Extract_orthologous_sequence_groups_from_assembled_contigs.py”, which is available online (https://github.com/zhangpenglab/UPrimer/tree/main/Accessory).

#### Phylogenetic Analyses

The nucleotide sequences of the 100 NPCLs were aligned based on their deduced protein sequences using MAFFT version 7.0.1 (Katoh and Standley 2013) with default settings. The resulting alignments were then refined using Gblocks version 0.91 (Castresana 2000) under codon mode (-t = c) with half gaps allowed (-b5 = h). To remove possible erroneous sequences in the alignments, maximum-likelihood (ML) trees were constructed for each refined NPCL alignment using IQ-Tree2 v. 2.1.0 (Minh et al. 2020); under the GTR + G model, unexpectedly long branches were eliminated using TreeShrink v. 1.3.3 (Mai and Mirarab 2018) with the false-positive error rate set to 0.05. Finally, the 100 filtered NPCL alignments were combined into a concatenated supermatrix, setting a 3-partition scheme with 1 partition for each codon position. ML inference was performed by IQ-Tree2, with 500 rounds of standard nonparametric bootstrapping replicates (-b 500) to assess clade support and using ModelFinder (Kalyaanamoorthy et al. 2017) to choose the best model for each partition via the option -MFP. To ensure the inferred trees were stabilizing, we repeated 3 independent IQ-TREE runs and obtained identical topologies and similar nodal support.

## Results

### UPrimer: Implementation of Automatically Designing NPCL Primers from Genome Data

UPrimer developed in this study is a bioinformatics tool that utilizes genomic data as input to automatically locate suitable NPCLs and design clade-specific nested-PCR amplification primers, without requiring any manual intervention. The software is written in Python 2.7 and runs on the command line under Linux. The outputs of UPrimer are a table of universal nested-PCR primers for many NPCLs, along with the corresponding reference DNA and protein sequences of these NPCLs. Each NPCL has 4 primers for nested-PCR amplification: a pair of inner forward/reverse primers, and a pair of outer forward/reverse primers. Our lab runs UPrimer on a Linux Centos 2.6.32 environment, with 2 Xeon (R) E5-2690 v3 CPUs (24 cores, 48 threads), and 256-GB RAM. A complete primer design analysis typically takes between 10 and 14 h to run. In summary, UPrimer provides a user-friendly and automated solution for designing clade-specific nested-PCR primers for NPCLs. It is freely available on GitHub online (https://github.com/zhangpenglab/UPrimer).

### The Newly Developed NPCL Primer Sets for 21 Metazoan Groups

We used UPrimer to develop 21 sets of universal nested-PCR primers of NPCLs for 21 metazoan groups, including Araneae, Sarcoptiformes, Hexanauplia, Decapoda, Entomobryomorpha, Blattodea, Lepidoptera, Coleoptera, Hymenoptera, Diptera, Heteroptera, Sternorrhyncha, Auchenorrhyncha, Bivalvia, Cephalopoda, Gastropoda, Actiniaria, Scleractinia, Cyclophyllidea, Echinodermata, and Vertebrata. The 21 newly designed primer sets each contained an average of ∼1,000 NPCLs, ranging from 328 to 2,316. The overall average length of the target NPCL regions was 721 bp, ranging from 556 to 828 bp across the 21 metazoan groups. The data flow details for the primer development process of each metazoan group are given in Table 1. The results of the NPCL primer set development, including the highest-scoring nested-PCR primer table and the reference sequences of the target NPCLs, are given in supplementary Appendices S1 to S21, Supplementary Material online. These newly developed NPCL primer sets are expected to be of great value for applying amplicon capture on these 21 metazoan groups.

When developing primer sets for Lepidoptera and Heteroptera, we tried 2 reference species with different genome data qualities. For Lepidoptera, *Bombyx mori* exhibited higher genome data quality (scaffold N50 = 12.2 Mb; BUSCO complete = 98.9%; exon number = 267,888) than *Danaus plexippus* (scaffold N50 = 9.2 Mb; BUSCO complete = 98.9%; exon number = 101,573). The number of amplicons in the primer table was 1,048 when using *B. mori* as the reference species, while the number decreased to 590 when using *D. plexippus* as the reference species (Table 1). A similar trend was observed in Heteroptera. *Cimex lectularius* showed higher genome data quality (scaffold N50 = 1.6 Mb; BUSCO complete = 99.4%; exon number = 250,049) than *Rhodnius prolixus* (scaffold N50 = 1.2 Mb; BUSCO complete = none; exon number = 89,655). When *C. lectularius* was replaced by *R. prolixus* as the reference species, the amplicon number of the developed primer table decreased from 526 to 328 (Table 1). These results show that selecting a reference species with high-quality genome data is beneficial for developing more NPCLs for a target group.

### PCR Performance Test of the NPCL Primers Designed by UPrimer

We conducted nested-PCR and standard-PCR amplification tests on 6 newly developed primer sets for Decapoda, Gastropoda, Vertebrata, Araneae, Heteroptera, and Lepidoptera. A total of 60 primer pairs were tested for the first 5 groups and 100 primer pairs for Lepidoptera. The amplification results of these 6 sets using 2 PCR strategies are shown in supplementary Fig. S1, Supplementary Material online. Overall, the success rate of amplification using the nested-PCR strategy is significantly higher compared with the standard-PCR strategy. The nested-PCR strategy achieved a PCR success rate of 95%, while the standard-PCR strategy only reached 65%. Additionally, there was a considerable proportion of reactions in that nested PCR produced strong target bands while standard-PCR produced nonspecific amplification, as depicted in supplementary Fig. S1, Supplementary Material online. These results emphasize the crucial need and importance of employing the nested-PCR strategy when designing NPCL primers.

**Fig. 4.**
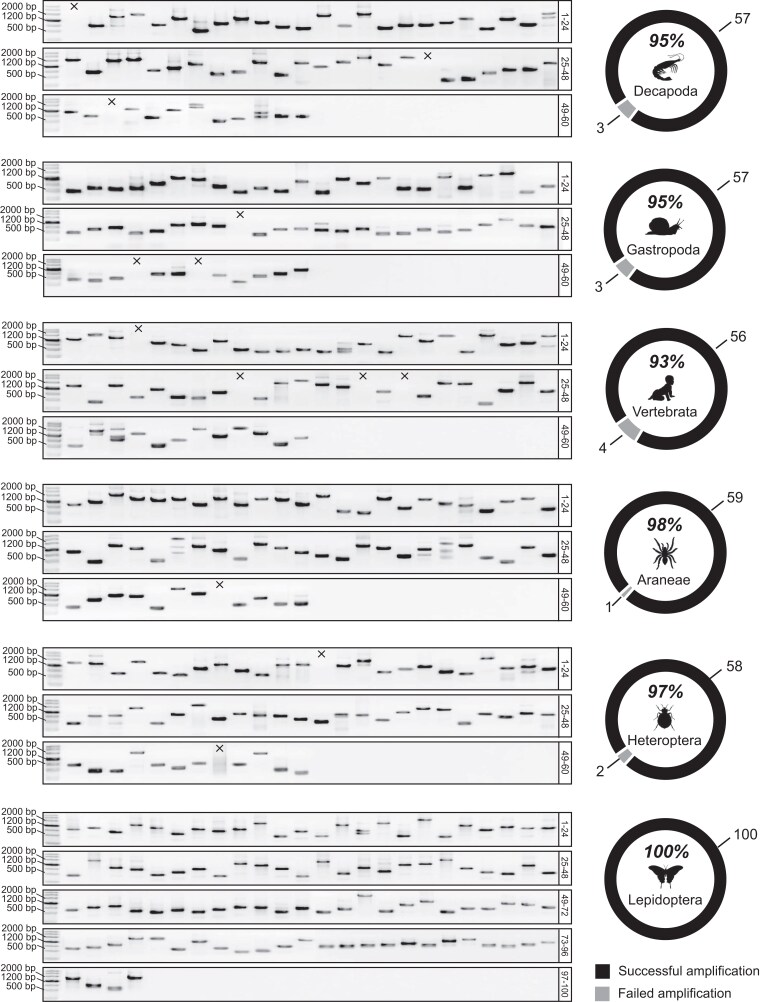
Results of amplification tests using newly developed NPCL primers for 6 metazoan groups (Decapoda, Gastropoda, Vertebrata, Araneae, Heteroptera, and Lepidoptera). Agarose gel electrophoresis results are shown on the left, and the overall amplification success is indicated on the right. Cross symbols denote the failure of NPCL amplification (no target band).

Figure 4 shows the detailed results of nested-PCR amplification tests for the 6 metazoan groups. The majority of the PCR reactions yielded a distinct single band of the anticipated size. Of the tested primer pairs in Decapoda, Gastropoda, Vertebrata, Araneae, Heteroptera, and Lepidoptera, 57, 57, 56, 59, 58, and 100 were successfully amplified, respectively. The success rate for each group was 95% (57/60), 95% (57/60), 93% (55/60), 98% (59/60), 97% (58/60), and 100% (100/100), indicating that the NPCL nested-PCR primers developed by UPrimer have a high success rate of amplification, exceeding 95% on average. Notably, our PCR experiments were conducted only once without any condition optimization, showing the ease of use of the NPCL primers developed by UPrimer. Although some amplification results shown in Fig. 4 have nonspecific amplification bands, our target bands are visible with the expected size. It should be mentioned that the presence of nonspecific bands in the amplification results will only decrease the efficiency of capturing the target regions with amplicon probes. As long as target NPCLs are present in the amplicon probes, these target NPCL sequences will be captured. Nonspecific amplification in the amplicon probes may also capture nontarget fragments in the libraries, but those nontarget sequences will be filtered out during the subsequent bioinformatics analysis and thus will not be included in the final phylogenomic analysis. In summary, the results of the 6 amplification experiments demonstrate that the universal primer sets for amplifying NPCLs developed by UPrimer not only are user-friendly but also have a high success rate of amplification. This feature improves the efficiency of collecting a large number of amplicons and making them into capture probes.

### The Performance of Applying Amplicon Capture in Lepidoptera

We amplified 100 NPCL fragments using mixed DNAs of lepidopteran samples and utilized the resulting amplicons to produce homemade probes. These probes were then used to capture target coding regions from 26 butterfly (Lepidoptera, Papilionoidea) and 7 outgroup moth samples. After sequencing and data quality control, we obtained a total of 344,262,468 clean 150 bp paired-end reads (∼51.6 Gb of data). The number of clean reads for each sample ranged from 3,894,540 to 23,677,898, with an average of 10 million reads (or 1.5 Gb of data) per sample. After contig assembling and orthologous sequence searching, our data processing results showed that all but 1 species (*Luehdorfia puziloi*) were able to recover ≥99 out of the 100 target NPCL sequences. We did not observe any significant differences in the number of target NPCL recovered between samples of ingroups and outgroups. This is likely due to the use of pooled amplicon probes generated by DNA mixtures. For detailed sequencing and assembly results of each sample, please refer to Table 2.

The read-to-target mapping percentage, also known as on-target rate, is commonly used to evaluate capture efficiency. The on-target rate of the 33 samples ranged from 20.56% to 57.01%, with an average of 36.31% (as shown in Table 2). The enrichment fold (EF) for amplicon capture was calculated using the formula: EF = on-target/(target size/genome size). The value of (target size/genome size) represents the percentage of fragments in the library that belong to the target regions, assuming that the DNA libraries were not enriched by hybridization capture. Using the average genome size of lepidopteran species (372 Mb) as a reference, the EF of using amplicon probes in the 33 samples ranged from 1,000-fold to 3,000-fold, with an average of approximately 1,911-fold.

Our homemade 100 NPCL amplicon probes were designed to capture approximately 71,400 bp of coding regions. The total length of target sequences recovered from all samples ranged from 55,917 to 71,133 bp, with an average of 67,947 bp (as shown in Table 2). This indicates that 95.1% of the target CDSs could be recovered for all captured samples. Interestingly, we observed a substantial amount of flanking sequences being captured through hitchhiking, most of which were CDSs useful for phylogenetic analysis. When these flanking CDSs were included, the average total capture length increased to 114,469 bp, which is 65.1% (46,520 bp) longer than expected. This result showed that most of the orthologous contigs contain flanking CDSs. However, we noticed that the length of flanking CDSs in some species, such as *L. puziloi*, *Pieris canidia*, and *Polycaena lua*, was obviously shorter than the average level. This difference should be attributed to the poor DNA quality of these samples (as shown in supplementary Table S3, Supplementary Material online), which resulted in smaller library inserts and thus constrained the amplicon probes from capturing longer flanking sequences.

### Phylogenetic Analysis

**Fig. 5.**
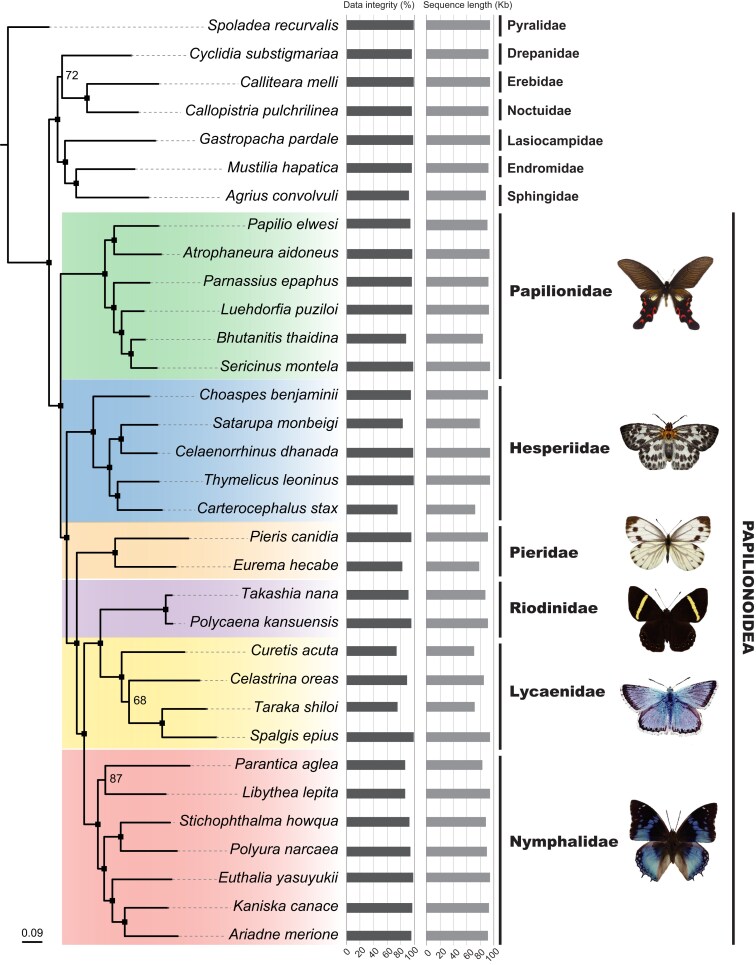
ML phylogeny of the 26 butterflies and 7 moths inferred from the 100 NPCL data set (∼98.8 K). The tree is inferred with IQ-Tree2. Values beside nodes are standard nonparametric BS support. The filled squares represent ML BS support = 100%. Two bars right to the species name represents the data integrity (calculated by nucleotides) for each species (1 minus the missing-data% of the species in the supermatrice) and the actual sequence length in the supermatrice for each species (excluding missing data “N” and gap “-“), respectively.

To fully utilize the data captured by amplicon probes, we included both the target NPCL regions and their flanking CDSs into our phylogenetic analysis. After sequence aligning and refining, the length of the 100 NPCL alignments ranged from 458 to 2,387 bp, with an average length of 988 bp. The concatenated data set of the 100 NPCL alignments is 98,834 bp in length. Data integrity for each species (1 minus the missing data % of the species in the supermatrice) ranged from 73.8% to 98.9%, with an average of 92.8% (more details on data integrity can be found in Fig. 5).

ML analyses on the concatenated data set produced a well-resolved phylogeny for the 26 sampled butterfly species, with 90% of nodes having 100% bootstrap (BS) support values (Fig. 5). The resulting backbone phylogeny of the butterflies and moths sampled in this study is consistent with other recent studies (e.g. Kawahara and Breinholt 2014; Breinholt et al. 2018; Kawahara et al. 2019). The monophyly of the Papilionoidea is strongly supported (BS = 100%, Fig. 5). Within the superfamily Papilionoidea, Papilionidae was recovered as the sister group to Hesperiidae + the rest of the butterflies with strong support (BS = 100%, Fig. 5), and Pieridae was strongly supported as the sister group to Nymphalidae and Lycaenidae + Riodinidae (BS = 100%, Fig. 5), consistent with many previous studies (Mutanen et al. 2010; Heikkilä et al. 2012; Regier et al. 2013; Kawahara and Breinholt 2014; Breinholt et al. 2018; Espeland et al. 2018). These results demonstrate that the NPCL amplicon probes developed by UPrimer contain substantial phylogenetic signal. A robust higher-level phylogeny of butterflies was reconstructed using only 100 amplicon probes, indicating the efficiency and effectiveness of amplicon capture.

## Discussion

### Choosing between Low-Coverage WGS and Sequence Capture: Considerations, and Suggestions

Sequence capture and low-coverage whole-genome sequencing (WGS) (e.g. Allen et al. 2017; Hughes and Teeling 2018; Allio et al. 2019; Zhang, Ding, et al. 2019; Ribeiro et al. 2021) are currently the 2 prevailing methods for acquiring phylogenomic data from nonmodel organisms. Low-coverage WGS involves sequencing the complete genome of an organism using short-read sequencing at a lower depth (∼5 to 10×) and utilizing de novo assembly to extract the targeted loci of interest. Theoretically, WGS data cover the entire genome, thereby granting researchers the capability to extract any desired loci. In contrast to sequence capture, low-coverage WGS does not demand high-quality genomic resources for designing probes for the target taxa, and it omits the hybridization enrichment step, making it a more executable method. Although low-coverage WGS has higher sequencing cost, this method is still generally more cost-effective than sequence capture, given that sequence capture uses expensive hybridization probes and the HTS sequencing cost has continued to decrease in recent years. However, low-coverage WGS is unsuitable for application in species with large genomes (e.g. >1 Gb), as de novo genome assembly from short-read data becomes considerably challenging with larger genome sizes, compromising the extraction of targeted loci (Zhang, Ding, et al. 2019). Another limitation is that processing WGS data requires significant computational resources and time, particularly when dealing with a large number of samples.

Sequence capture is designed to enrich specific target regions of the genome, thus only requiring fewer sequencing data (less than 1× coverage of genome) to achieve a high sequencing depth within these regions. This feature renders sequence capture less sensitive to genome sizes. Moreover, the processing of capture data is much easier than that of WGS data, demanding fewer computational resources and less time. For instance, assembling low-coverage WGS data (5 to 10×) for 10 species within the Eudaminae family (genome size: 500 to 600 Mb) took several to dozens of hours and utilized several dozens of gigabytes of memory, while assembly of sequence capture data only took a few minutes to several dozen minutes, and required only a few gigabytes of memory (G Ribeiro et al. 2021). Consequently, sequence capture is well suited for large-scale phylogenomic studies.

Considering the characteristics and applicability of these 2 methods, we offer the following suggestions to aid researchers in choosing the suitable approach for acquiring phylogenomic data: (i) for situations where the target taxa possess small genomes (<600 Mb) and the sample size remains modest (<200 samples), low-coverage WGS sequencing (∼5 to 10× coverage) is a good choice. (ii) In cases where the target taxa exhibit relatively large genomes (>1 Gb) or the project encompasses a substantial number of samples (200 to 500 or more), sequence capture generally holds a greater advantage.

### Merits of UPrimer

UPrimer is a highly efficient and user-friendly tool that enables the screening of hundreds to thousands of NPCLs from genome data and the design of universal amplification primers in a high-throughput manner. The tool only requires the user to provide FASTA-formatted files of the relevant species’ genome and type a single command line to invoke UPrimer. Subsequently, the program automatically processes the data and generates a primer table. UPrimer is fully automated and can perform all steps involved in universal primer design. In contrast, other similar bioinformatics tools are all semiautomated. For example, MarkerMiner (Chamala et al. 2015) and DOMINO (Frías-López et al. 2016) can only construct MSAs from NGS, genome, or transcriptome data but do not design primers; PRIMERVIEW (O'Halloran 2015) and DISCOMARK (Rutschmann et al. 2017) can design primers in a high-throughput manner but require users to provide precomputed MSAs. Consequently, UPrimer’s fully automated design process makes it especially suitable for users without extensive bioinformatics and primer design experience.

Another main difference with other primer design programs of UPrimer is that it adopts the nested-PCR strategy to develop amplification primers for NPCLs. This change greatly increases the amplification success rate of the designed primers and reduces the experimental labor intensity. Users do not need to optimize amplification conditions for each NPCL but simply perform a large number of PCRs in 96-well plates using the same amplification condition. This feature is crucial for preparing amplicon probes in a large scale. Our PCR amplification test of 6 metazoan primer sets suggested that, whatever at the level of suborder (Heteroptera), order (Araneae, Lepidoptera), class (Gastropoda), or subphylum (Vertebrata), the success rate of PCR amplification for nested-PCR primers designed by UPrimer is generally more than 90%. Such a high PCR success rate means that users can directly order the primers from the primer table generated by UPrimer to prepare amplicon probes without the need of pre-amplification experiments and optimizing amplification conditions.

### Suggestions for Using UPrimer to Develop Clade-Specific NPCL Primers

The final number of NPCLs is a crucial indicator to determine the success or failure of the universal primer set development. A large number of workable NPCLs (at least 100) are necessary for effectively applying amplicon capture in phylogenomic studies. To successfully develop a set of clade-specific NPCL primers using UPrimer, 4 aspects require special attention:

Firstly, the selection of an appropriate reference species is crucial for obtaining more NPCL primers. The reference species should represent the targeted clade and possess a high-quality genome and annotations to facilitate the identification of a greater number of long- and single-copy exonic loci. Taking Lepidoptera as an example, the genome and annotation quality of *B. mori* are superior to those of *D. plexippus*. As a result, selecting *B. mori* as the reference species for primer design resulted in 44% more NPCL primers than using *D. plexippus* (Table 1).

Secondly, there should be adequate ingroup species representation. To ensure that the designed NPCL primers are universal across the targeted clade, each major linage of the target clade should have at least 1 representative ingroup species included in the analysis, and the total number of ingroups is relatively moderate (10 to 15). This will help minimize the bias in locating conserved primer blocks and increase the accuracy of NPCL primer design.

Thirdly, the inclusion of outgroups can enhance the credibility of discovering conserved primer blocks and increase the PCR success rate of the designed primers. However, improper utilization of outgroups may impact the number of the ultimate NPCL outputs. When no closely related taxa are available as outgroups for the target clade, introducing distantly related ones may pose difficulties in identifying orthologous sequences, obtaining a sufficient number of candidate MSAs, and identifying conserved primer blocks. This is why we did not include outgroups in the design of NPCL primers for Echinodermata and Vertebrata (see Table 1).

Lastly, validation of the clade-specific NPCL primers is necessary to assess their amplification efficiency and specificity. This step involves testing ∼50 NPCL primer sets by PCR and verifying the resulting amplicons through electrophoresis. A PCR success rate of more than 90% is anticipated. It should be noted that, in our experience, the presence of significant nonspecific bands in the amplification product does not impede successful amplicon capture, provided that the target bands are present.

### New NPCL Primer Resource for Amplicon Capture Phylogenomics of Metazoans

In this study, we have developed clade-specific NPCL primer sets for 21 metazoan groups that can be utilized for amplicon capture phylogenomics (as shown in Table 1). Among these metazoan groups, 8 (namely, the phylum Echinodermata, the class Bivalvia, Cephalopoda, Gastropoda of Mollusca, the class Hexanauplia, the order Cyclophyllidea of Platyhelminthes, and the order Blattaria and Entomobryomorpha of Arthropoda) lack usable AHE/UCE probes (for available AHE/UCE probe resources, see supplementary Table S4 in Supplementary Material online). The development of these clade-specific NPCL primer sets provides a novel resource for the phylogenomic analysis of metazoan groups, especially those without usable AHE/UCE probes. By utilizing these NPCL primers for amplicon capture sequencing, researchers can obtain comparable sequence data with traditional AHE/UCE sequencing, making this method a valuable supplement to the latter. Based on our experience, a typical data set captured by 500 to 1000 NPCL amplicon probes for these groups comprises approximately 500-kb to 1-Mb DNA sequences, which is sufficient for phylogenomic inference of most animal groups. Our case study in Lepidoptera demonstrates the feasibility and effectiveness of using NPCL primers for amplicon capture sequencing and phylogenomic analyses.

In summary, this study provides a valuable contribution to the field of metazoan phylogenomics by offering new NPCL primer resources for amplicon capture sequencing and demonstrating the effectiveness of this method. These resources and our case study can serve as a reference for future researchers who aim to conduct phylogenomic studies in metazoans.

### Amplicon Capture Is a Promising Method for Phylogenomic Analyses

Experimental cost and time are 2 important considerations when using target sequence capture for phylogenomic research. Amplicon capture is a cost-effective and time-saving method since the preparation of amplicon probes can be done by the user in the laboratory. Each NPCL requires 4 amplification primers with a total length of 86 nucleotides. The total cost of primer synthesis for 1 NPCL is 25.8 CNY (using a 5.0-nmole package with HAP purity from Sangon Inc., Shanghai, China). Taking into account the amplification expense, the total cost of preparing 1 amplicon probe is less than 30 CNY. Preparing a set of amplicon probes containing 500 target NPCLs costs approximately 15,000 CNY, and 1 person can complete probe preparation at this scale within 3 to 5 d using a single 96-well thermocycler. On the other hand, ordering a 24-reaction AHE or UCE probe kit typically costs between $2,500 and $5,000 (equivalent to 17,500–35,000 CNY) and takes several weeks to months, depending on the supplier. When handling a large-scale project with hundreds of samples, using commercial probes will incur higher costs as more probe kits need to be ordered, whereas the use of amplicon probes is nearly free except for the initial primer investment. Furthermore, after NPCL primers are synthesized, they can be reused for lower taxonomic unit within their initial target taxon, which will reduce its use cost further.

Compared with commercial probe kits, amplicon capture offers greater flexibility in probe preparation, as the number of amplicon probes can be adjusted to meet the data requirements of a specific project. For example, if one aims to resolve the phylogeny of a lower taxon such as a family, tribe, or genus, using 100 to 200 NPCL amplicon probes may suffice. However, for higher-level phylogenetic questions (e.g. family-level phylogeny), using 200 to 1,000 NPCL amplicon probes may be required. Amplicon capture provides a controllable solution for collecting phylogenomic data. In contrast, commercial probe kits are limited by their original design, and their probe composition cannot be adjusted after synthesis.

Like AHE/UCE sequencing, amplicon capture has wide applicability in phylogenetic research, allowing for investigations of phylogenetic questions ranging from deep to shallow divergences. A single set of clade-specific NPCL primers can resolve phylogenetic relationships at different taxonomic levels. For example, the Lepidoptera NPCL primer set has been shown to effectively reconstruct robust phylogenetic relationships within various Lepidoptera groups, such as the superfamily Papilionoidea (using 100 NPCL probes; this study), the family Epicopeiidae (using 150 NPCL probes; Zhang et al. 2020), and the genus *Neptis* (using 150 NPCL probes; Ma et al. 2020). Furthermore, the average sequence length of a target locus from amplicon capture (∼1,000 bp) is typically longer than that of AHE/UCE sequencing (300 to 600 bp). Longer loci provide a stronger phylogenetic signal and are less prone to GTEE, resulting in more accurate gene tree estimation, which is highly beneficial for multispecies coalescence species tree reconstruction (Salichos and Rokas 2013; Roch and Warnow 2015; Karin et al. 2020).

Compared with the commonly used AHE/UCE sequencing, amplicon capture offers several advantages, including cost-effectiveness, experimental flexibility, and the ability to obtain longer gene loci. Therefore, amplicon capture is a promising method for collecting phylogenomic data to accelerate resolving various parts of the Tree of Life.

### Future Directions

In the future, the amount of genomic data from various organisms will continue to increase at a rapid pace, providing ample opportunities for the development of more clade-specific NPCL primer sets. This will expand the applicability of amplicon capture technology in phylogenetic studies to encompass a broader range of biological taxa. Furthermore, there is potential for further improvement in UPrimer. Specifically, (i) integrating visualization and online design features and (ii) implementing primer design on cloud platforms to remove the necessity of downloading genomic resources onto individual servers. This would be particularly advantageous for teams with limited computational resources.

## Conclusion

UPrimer is a user-friendly software tool designed to automatically develop clade-specific NPCL amplification primers from genomic data, without the need for manual intervention. It adopts a nested-PCR amplification strategy for high success rate gene amplification and can generate universal primer sets for hundreds to thousands of NPCLs with just a single command line. Our evaluation of UPrimer in 6 metazoan groups has demonstrated a success rate of at least 90% for gene amplification of its designed primers. The successful phylogenetic analysis of butterflies using UPrimer's developed NPCL primers further demonstrates its effectiveness. We provided a detailed protocol on how to perform a typical amplicon capture experiment, including amplicon bait preparation, sequence capture, and data analysis. The UPrimer program and the amplicon capture experimental protocol are freely available on GitHub online (https://github.com/zhangpenglab/UPrimer).

## Supplementary Material

msad230_Supplementary_Data

## Data Availability

Raw read data for 33 Lepidoptera samples were deposited in NCBI SRA (accession PRJNA956657). The refined alignments of 100 NPCLs, the final DNA concatenated data matrix, and the resulting phylogenetic tree output from IQ-Tree2 are deposited in Figshare (https://figshare.com/s/3a648997a162eb70a5a7).
