# Correction to: UPrimer: A Clade-Specific Primer Design Program Based on Nested-PCR Strategy and Its Applications in Amplicon Capture Phylogenomics

**DOI:** 10.1093/molbev/msaf317

**Published:** 2025-12-09

This is a correction to: JiaXuan Li, GuangCheng Han, Xiao Tian, Dan Liang, Peng Zhang, UPrimer: A Clade-Specific Primer Design Program Based on Nested-PCR Strategy and Its Applications in Amplicon Capture Phylogenomics, *Molecular Biology and Evolution*, Volume 40, Issue 11, November 2023, msad230, https://doi.org/10.1093/molbev/msad230

This is a correction to the supplementary data for this article. The below supplementary files have been updated to revise the reference datasets corresponding to the newly developed NPCL primer sets for 21 metazoan groups. These reference sequences are essential for the subsequent analysis of amplicon capture data, as they are used to identify and extract the target genomic regions from sequencing data.

These details have been corrected only in this correction notice to preserve the published version of record.

