# Supplementary material for: UPrimer: A Clade-Specific Primer Design Program Based on Nested-PCR Strategy and Its Applications in Amplicon Capture Phylogenomics
Source: Mol Biol Evol. 2023 Oct 13;40(11):msad230. doi: 10.1093/molbev/msad230 (PMC10630340; doi:10.1093/molbev/msad230)
Supplement: msad230_Supplementary_Data [file msad230_supplementary_data.zip › Figure S1.pdf]

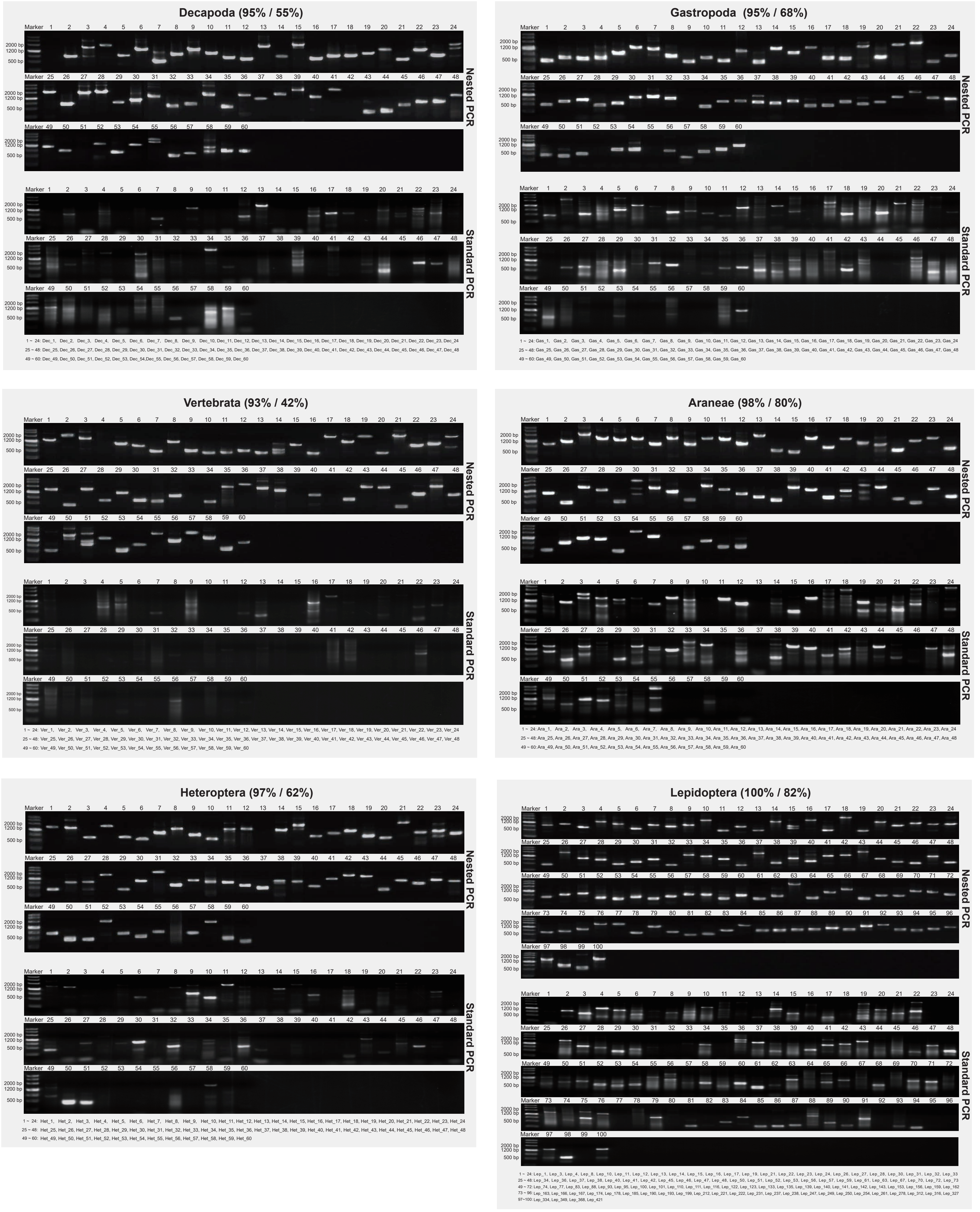

Figure S1. Agarose gel electrophoresis results of nested PCR and standard PCR amplification using newly developed NPCL primers for six metazoan groups (Decapoda, Gastropoda, Vertebrata, Araneae, Heteroptera, and Lepidoptera). The overall amplification success rate is indicated on the right of the group name (left: nested PCR, right: standard PCR).
