# Supplementary material for: UPrimer: A Clade-Specific Primer Design Program Based on Nested-PCR Strategy and Its Applications in Amplicon Capture Phylogenomics
Source: Mol Biol Evol. 2023 Oct 13;40(11):msad230. doi: 10.1093/molbev/msad230 (PMC10630340; doi:10.1093/molbev/msad230)
Supplement: msad230_Supplementary_Data [file msad230_supplementary_data.zip › Supplementary Table S4. List of publicly available probe resources of UCE and AHE sequencing.docx]

| **Type** | **Name of probe set** | **Clade** | **Number of loci** | **Average locus length (bp)** | **Reference** |
| --- | --- | --- | --- | --- | --- |
| UCE | Tetrapods-UCE-2.5kv1 | **Chordata: Tetrapoda** | 2386 | 93 | Faircloth et al. 2012 |
|  | Actinopterygians 0.5Kv1 | **Chordata: Actinopterygii** | 500 | 120 | Faircloth et al. 2013 |
|  | Acanthomorphs 1Kv1 | **Chordata: Acanthomorpha** | 1,314 | unknown | Alfaro et al. 2013 |
|  | Arachnida 1.1Kv1 | **Arthropoda: Arachnida** | 1,120 | unknown | Faircloth 2017 |
|  | Spider-Specific | **Arthropoda: Araneae** | 2,021 | unknown | Kulkarni et al. 2020 |
|  | Coleoptera 1.1Kv1 | **Arthropoda: Coleoptera** | 1,172 | unknown | Faircloth 2017 |
|  | Diptera 2.7Kv1 | **Arthropoda: Diptera** | 2,711 | unknown | Faircloth 2017 |
|  | Hemiptera 2.7Kv1 | **Arthropoda: Hemiptera** | 2,731 | unknown | Faircloth 2017 |
|  | Lepidoptera 1.3K-v1 | **Arthropoda: Lepidoptera** | 1,381 | unknown | Faircloth 2017 |
|  | Hymenoptera 1.5Kv1 | **Arthropoda: Hymenoptera** | 1,510 | unknown | Faircloth et al. 2015 |
|  | Anthozoa 1.7Kv1 | **Cnidaria: Anthozoa** | 720 | 307 | Quattrini et al. 2018 |
|  | / | **Platyhelminthes: Schistosomatidae** | ~4000 | 349 | Ebbs et al. 2022 |
|  |  |  |  |  |  |
| AHE | AHE | **Chordata** | 512 | 240 | Lemmon et al.2012 |
|  | Beetle | **Arthropoda: Coleoptera** | 941 | 440 | Haddad et al. 2017 |
|  | Diptera | **Arthropoda: Diptera** | 546 | 588 | Young et al. 2016 |
|  | / | **Arthropoda: Hemiptera** | 514 | 296 | Dietrich et al. 2017 |
|  | Hym_Ich | **Arthropoda: Hymenoptera** | 541 | 392 | Baker et al. 2020 |
|  | / | **Arthropoda: Trichoptera** | 989 | 232 | Deng et al. 2021 |
|  | LEP1 | **Arthropoda: Lepidoptera** | 855 | 254 | Breinholt et al. 2017 |
|  | BUTTERFLY1.0 | **Arthropoda: Lepidoptera** | 425 | 430 | Espeland et al. 2018 |
|  | / | **Arthropoda: Decapoda** | 410 | 209 | Wolfe et al. 2019 |
|  | Unioverse | **Mollusca: Unionidae** | 811 | 214 | Pfeiffer et al. 2019 |

**Table S4.** List of publicly available probe resources of UCE and AHE sequencing.

**Reference**

Alfaro ME, Faircloth BC, Harrington RC, Sorenson L, Friedman M, Thacker CE, Oliveros CH, Černý D, Near TJ. 2018. Explosive diversification of marine fishes at the Cretaceous-Palaeogene boundary. *Nat Ecol Evol.* 2(4):688–696.

Baker AJ, Heraty JM, Mottern J, Zhang J, Hines HM, Lemmon AR. 2020. Inverse dispersal patterns in a group of ant parasitoids (Hymenoptera: Eucharitidae: Oraseminae) and their ant hosts. *Syst Entomol.* 45(1):1–19.

Breinholt JW, Earl C, Lemmon AR, Lemmon EM, Xiao L, Kawahara AY. 2018. Resolving relationships among the megadiverse butterflies and moths with a novel pipeline for anchored phylogenomics. *Syst Biol.* 67(1):78–93.

Deng XL, Favre A, Lemmon EM, Lemmon AR, Pauls SU. 2021. Gene Flow and Diversification in Himalopsyche martynovi Species Complex (Trichoptera: Rhyacophilidae) in the Hengduan Mountains. *Biology (Basel)*. 10(8):816.

Dietrich CH, Allen JM, Lemmon AR, Lemmon EM, Takiya DM, Evangelista O, Walden KK, Grady PG, Johnson KP. 2017. Anchored hybrid enrichment-based phylogenomics of leafhoppers and treehoppers (Hemiptera: Cicadomorpha: Membracoidea). *Insect Syst Divers*. 1(1):57–72.

Ebbs ET, Loker ES, Bu L, Locke SA, Tkach VV, Devkota R, Flores VR, Pinto HA, Brant SV. 2022. Phylogenomics and Diversification of the Schistosomatidae Based on Targeted Sequence Capture of Ultra-Conserved Elements. *Pathogens*. 11(7):769.

Espeland M, Breinholt J, Willmott KR, Willmott KR, Warren AD, Vila R, Toussaint EFA, Maunsell SC, Aduse-Poku K, Talavera G, Eastwood R, Jarzyna MA, Guralnick R, et al. 2018. A Comprehensive and Dated Phylogenomic Analysis of Butterflies. *Curr Biol.* 28(5):770–778.

Faircloth BC, McCormack JE, Crawford NG, Harvey MG, Brumfield RT, Glenn TC. 2012. Ultraconserved elements anchor thousands of genetic markers spanning multiple evolutionary timescales. *Syst Biol.* 61(5):717–726.

Faircloth BC, Sorenson L, Santini F, Alfaro ME. 2013. A Phylogenomic Perspective on the Radiation of Ray-Finned Fishes Based upon Targeted Sequencing of Ultraconserved Elements (UCEs). *PLoS One.* 8(6):e65923.

Faircloth BC, Branstetter MG, White ND, Brady SG. 2015. Target enrichment of ultraconserved elements from arthropods provides a genomic perspective on relationships among Hymenoptera. *Mol Ecol Resour.* 15(3):489–501.

Faircloth BC. 2017. Identifying conserved genomic elements and designing universal bait sets to enrich them. *Methods Ecol Evol.* 8(9):1103–1112.

Haddad S, Shin S, Lemmon AR, Lemmon EM, Svacha P, Farrell B, Slipinski A, Windsor D, McKenna DD. 2018. Anchored hybrid enrichment provides new insights into the phylogeny and evolution of longhorned beetles (Cerambycidae). *Syst Entomol.* 43: 68–89.

Kulkarni S, Wood H, Lloyd M, Hormiga G. 2020. Spider-specific probe set for ultraconserved elements offers new perspectives on the evolutionary history of spiders (Arachnida, Araneae). *Mol Ecol Resour.* 20(1):185–203.

Lemmon AR, Emme SA, Lemmon EM. 2012. Anchored hybrid enrichment for massively high-throughput phylogenomics. *Syst Biol.* 61(5):727–744.

Pfeiffer JM, Breinholt JW, Page LM. 2019. Unioverse: A phylogenomic resource for reconstructing the evolution of freshwater mussels (Bivalvia, Unionoida). *Mol Phylogenet Evol*. 137:114–126.

Quattrini AM, Faircloth BC, Dueñas LF, Bridge TCL, Brugler MR, Calixto-Botía IF, DeLeo DM, Forêt S, Herrera S, Lee S, et al. 2018. Universal target-enrichment baits for anthozoan (Cnidaria) phylogenomics: New approaches to long-standing problems. *Mol Ecol Resour.* 18(2):281–295.

Wolfe JM, Breinholt JW, Crandall KA, Lemmon AR, Lemmon EM, Timm LE, Siddall ME, Bracken-Grissom HD. 2019. A phylogenomic framework, evolutionary timeline and genomic resources for comparative studies of decapod crustaceans. *Proc Biol Sci*. 286(1901):20190079.

Young AD, Lemmon AR, Skevington JH, Mengual X, Ståhls G, Reemer M, Jordaens K, Kelso S, Lemmon EM, Hauser M, et al. 2016. Anchored enrichment dataset for true flies (order Diptera) reveals insights into the phylogeny of flower flies (family Syrphidae). *BMC Evol Biol*. 16(1):143.
